# Supplementary material for: A New Approach for the Development and Optimization of Gluten-Free Noodles Using Flours from Byproducts of Cold-Pressed Okra and Pumpkin Seeds
Source: Foods. 2023 May 16;12(10):2018. doi: 10.3390/foods12102018 (PMC10216911; doi:10.3390/foods12102018)
Supplement: Supplementary file 1 [file foods-12-02018-s001.zip › foods-2367821-supplementary.pdf]

Table S1 Regression and standardized regression coefficients of developed models for the construction of optimization problem.

Explanatory Variable	Symbol	z_{Col}	z_{ColH}	z_{Ar}	$z_{CookH.}$	z_{Chew}	z_{Stick}	$z_{PT.}$	z_{UPT}	z_{OA}
Constant	β_0	-3529.37***	4478.85***	-674.27***	-2446.59***	-2139.56***	3180.06***	-415.33*	-4613.85***	-769.94***
Okra seed (*OSF*)	B_{OSF}	204.09 [4407.18]***	-248.84 [-5950.37]***	39.67 [563.73]***	139.93 [3031.10]***	121.95 [2363.82]***	-167.89 [-2478.39]***	24.67 [353.57]*	261.73 [3773.83]***	46.90 [1159.89]***
Cold pressed pumpkin seed (*PSF*)	β_{PSFo}	204.28 [4281.23]***	-249.06 [-5780.02]***	39.74 [548.15]***	140.04 [2944.14]***	122.05 [2296.12]***	-167.94 [-2406.05]***	24.74 [344.08]*	262.06 [3667.22]***	46.97 [1127.54]***
Salt (*s*)	β_s	24.64 [4.31]***		8.40 [0.97]***	11.96 [2.10]***	12.67 [1.99]***	8.93 [1.07]***	6.91 [0.80]**	17.21 [2.01]***	11.30 [2.26]***
Water (*w*)	β_w	17.84 [22.96]***	-55.93 [-79.69]***	1.24 [1.05]***	18.13 [23.40]***	16.75 [19.34]***	-68.01 [-59.83]***		39.81 [34.20]***	-3.73 [-5.50]•
Gluten-free flour (*gff*)	β_{gff}	-92.53 [-2391.81]***	113.07 [3242.02]***	-17.84 [-304.05]***	-63.48 [-1648.82]***	-55.27 [-1284.50]***	76.57 [1355.23]***	-11.05 [-189.82]*	-118.62 [-2050.88]***	-21.14 [-626.84]***
OSF x *OSF*	$(\beta_{OSFs})^2$	-2.72 [-2708.47]***	3.32 [3660.00]***	-0.53 [-346.75]***	-1.86 [-1863.95]***	-1.63 [-1453.62]***	2.24 [1525.65]***	-0.33 [-217.57]*	-3.49 [-2266.99]***	-0.62 [-712.55]***
PSF x *PSF*	$(\beta_{PSF})^2$	-2.72 [-2645.02]***	3.33 [3575.16]***	-0.53 [-338.59]***	-1.87 [-1819.47]***	-1.63 [-1419.06]***	2.24 [1488.95]***	-0.33 [-212.48]*	-3.50 [-22.66]***	-0.63 [-695.96]***
w x *w*	$(\beta_w)^2$	-0.62 [-17.93]**	2.33 [55.07]***		-0.70 [-20.30]***	-0.63 [-16.55]***	2.95 [58.76]***	0.04 [0.82]***	-1.56 [-30.40]***	0.23 [7.51]*
gff x *gff*	$(\beta_{gff})^2$	3.21 [4042.14]***	-3.92 [-5461.65]***	0.62 [513.94]***	2.20 [2780.58]***	1.92 [2167.04]***	-2.65 [-2279.20]***	0.38 [321.28]*	4.12 [3459.34]***	0.74 [1060.20]***
OSF x *PSF*	$\beta_{OSF}\beta_{PSFo}$	-5.44 [-1612.26]***	6.64 [2179.20]***	-1.06 [-205.96]***	-3.73 [-1109.45]***	-3.25 [-865.11]***	4.48 [908.39]***	-0.66 [-129.08]*	-6.98 [-1381.93]***	-1.25 [-423.90]***
Model significance		***	***	***	***	***	***	***	***	***
R^2		0.91	0.97	0.98	0.95	0.95	0.97	0.97	0.90	0.98
R^2_{adj}		0.90	0.96	0.98	0.95	0.95	0.96	0.97	0.89	0.98
R^2_{pred}		0.88	0.96	0.98	0.94	0.94	0.96	0.96	0.89	0.98

- The given subscripts of z_i terms are Col: color, ColH: color homogeneity, Ar: Aroma, CookH: cooking homogeneity, Chew: chewiness, Stick: stickiness, PT: pleasant taste, UPT: unpleasant taste, and OA: overall acceptability.
- β_0 is the model constant (intercept), β_i is the linear coefficient (main effect), β_{ii} is the quadratic coefficient, and β_{ij} is the two factors interaction coefficient in adjusted polynomial model $z = \beta_0 + \sum_{i=1}^{3} \beta_i x_i + \sum_{i=1}^{3} \beta_{ii} x_i^2 + \sum_{i=1}^{2} \sum_{j=i+1}^{3} \beta_{ij} x_i x_j$. All coefficients were given as "regression coefficient [standardized regression coefficient]".
- R^2: Variability explained, R^2_{adj}: Variability explained adjusted, R^2_{pred}: Variability explained predicted.
- Significance codes (p): 0 < ' *** '< 0.001 < ' ** ' < 0.01 < ' * ' < 0.05 < ' • ' < 0.1 < ' ' < 1
